# The Serum Treatment of Fever after Delivery

**Published:** 1899-08-12

**Authors:** 


### THE SERUM TREATMENT OF FEVER AFTER DELIVERY.

In discussing at Portsmouth the serum treatment of fever following delivery, Dr. Herbert Spencer said that as usually applied serumtherapy in the treatment of puerperal fever had no scientific basis; that it had not lowered the mortality; that it usually lowered the temperature and improved the general condition; and that its use was not free from danger. In the discussion which followed Professor Byers, of Glasgow, after pointing out that, notwithstanding the progress of antiseptic principles, puerperal fever was not diminishing, showed an instrument for obtaining the secretions from the uterine cavity in a sterilised tube for bacteriological examination, and emphasised the fact that it was of no use to administer the serum without first demonstrating the presence of the streptococci. The real key to treatment was, however, indicated by Dr. Murdoch Cameron, of Glasgow, who brought the question of the management of a septic fever after a confinement round again to ordinary surgical principles, and showed the importance in such cases of emptying the uterus, and so removing what, in a very large proportion of the cases, was certain to be the origin of the infection. On the whole it was an interesting and useful discussion, and its drift was in the direction of common sense.

